# Sepia, Tarsier, and Chameleon: A Modular C++ Framework for Event-Based Computer Vision

**DOI:** 10.3389/fnins.2019.01338

**Published:** 2020-01-08

**Authors:** Alexandre Marcireau, Sio-Hoi Ieng, Ryad Benosman

**Affiliations:** ^1^INSERM UMRI S 968, Sorbonne Universites, UPMC Univ Paris 06, UMR S 968, CNRS, UMR 7210, Institut de la Vision, Paris, France; ^2^University of Pittsburgh Medical Center, Pittsburgh, PA, United States; ^3^Robotics Institute, Carnegie Mellon University, Pittsburgh, PA, United States

**Keywords:** silicon retinas, event-based sensing, development framework, event-based processing, asynchronous computation

## Abstract

This paper introduces an new open-source, header-only and modular C++ framework to facilitate the implementation of event-driven algorithms. The framework relies on three independent components: *sepia* (file IO), *tarsier* (algorithms), and *chameleon* (display). Our benchmarks show that algorithms implemented with *tarsier* are faster and have a lower latency than identical implementations in other state-of-the-art frameworks, thanks to static polymorphism (compile-time pipeline assembly). The *observer pattern* used throughout the framework encourages implementations that better reflect the event-driven nature of the algorithms and the way they process events, easing future translation to neuromorphic hardware. The framework integrates drivers to communicate with the *DVS*, the *DAVIS*, the *Opal Kelly ATIS*, and the *CCam ATIS*.

## 1. Introduction

Event-based cameras are fundamentally different from conventional cameras (Posch et al., 2014). Conventional, frame-based cameras integrate light at fixed time intervals, and produce spatially dense frames. By contrast, the pixels of event-based sensors are asynchronous and independent. Each pixel outputs data only when the visual information in its field of view changes, mimicking biological sensing (Liu and Delbruck, 2010). Event-based cameras output their events in the order they are produced, resulting in a spatially sparse sequence with sub-millisecond precision. This fundamental difference in data nature calls for different computational strategies (Delbruck et al., 2010).

This work introduces an end-to-end framework for designing and running event-based algorithms for computer vision. The code components are written in C++, and are open-source. The presented method and implementation outperform state-of-the-art frameworks while encouraging better semantics for event-based algorithms. Special care is given to modularity and code dependencies management, in order to facilitate portability and sharing.

### 1.1. Event-Based Cameras

Bio-inspired cameras aim at mimicking biological retinas, as the latter greatly outperform conventional, frame-based systems (Liu and Delbruck, 2010). Many architectures have been implemented over the years, including pulse-modulation imaging (Chen et al., 2011), smart vision chips (Dudek and Hicks, 2005; Carmona-Galán et al., 2013), and event-based sensors. The framework presented in this paper primarily targets event-based sensors.

**Figure 1:**
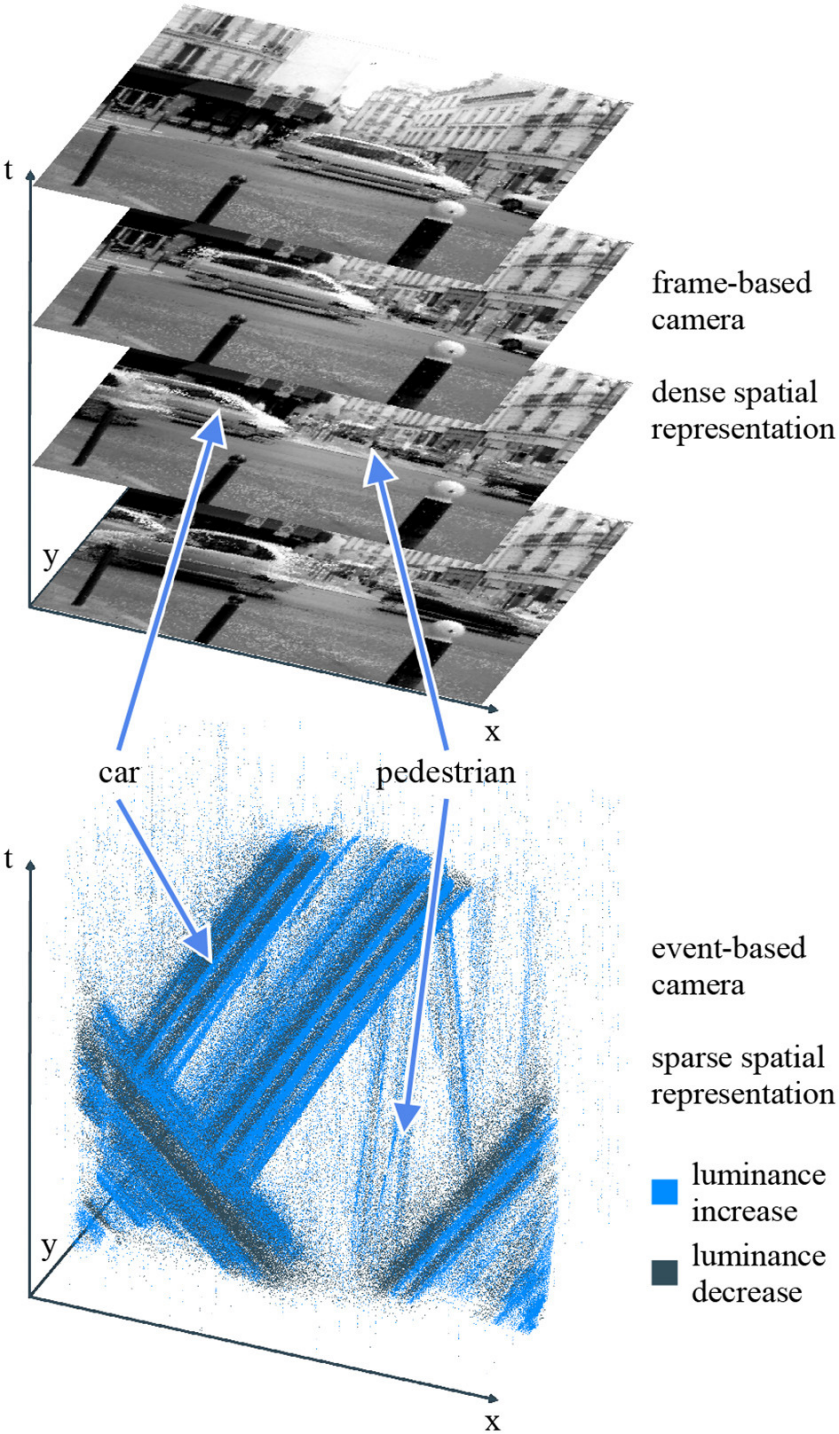
Conventional cameras (top) capture dense frames at fixed time intervals. Event-based cameras (bottom) have independent pixels which asynchronously output information when the luminance in their individual field of view changes. This sparse representation yields a better temporal resolution and a smaller bandwidth. Some computer vision tasks, such as moving objects segmentation, become easier. The point cloud representation is a still frame of a standalone HTML widget generated by our framework.

The pixels of event-based sensors contain analog circuits implementing signal processing calculations. Upon meeting a specific condition, the analog circuit emits an output transmitted to the computer. The most widespread type of calculation is brightness change detection. The pixel's photodiode output is continuously monitored to detect significant variations. When the logarithmic luminance changes beyond a fixed threshold, the pixel sends an event to the computer. This event bundles spatial and temporal information, as well as a boolean polarity encoding whether the significant change corresponds to an increase or decrease in brightness. Several sensors contain pixels implementing this behavior, including the *DVS* (Dynamic Vision Sensor) (Lichtsteiner et al., 2008), the *cDVS* (Berner and Delbruck, 2011), the *ATIS* (Asynchronous Time-based Image Sensor) (Posch et al., 2010), and the *DAVIS* (Dynamic and Active-pixel Vision Sensor) (Brandli et al., 2014). They are still under active development, with improved versions featuring lower latency (Lenero-Bardallo et al., 2011), higher sensitivity (Delbruck and Berner, 2010; Serrano-Gotarredona and Linares-Barranco, 2013; Yang et al., 2015), or more pixels (Son et al., 2017). Figure 1 highlights the difference between a sequence of frames and a stream of polarity events recorded from the same scene.

The *cDVS* and *ATIS* differ from the *DVS* by their extended pixel circuits generating a second type of polarity events, besides change detection. The polarity bit of the second event type encodes another visual information. The *cDVS* triggers such events on wavelength changes, whereas the *ATIS* encodes absolute exposure measurements in the time difference between them. The DAVIS is a hybrid sensor: it features both a *DVS*-like circuit and a light integration circuit. The latter produces frames similar to those generated by a conventional sensor. Huang et al. (2017) present another event-based sensor, the *CeleX*, with a behavior similar to that of a *DVS*: events are triggered by brightness changes. However, output events include an absolute exposure measurement encoded on 9 bits instead of a binary spolarity.

### 1.2. Event-Based Computer Vision

There are three approaches to information extraction from the output of event-based cameras. The first one consists in generating spatially dense frames from the sensor output in a way that preserves temporal resolution. The frames can then be fed to conventional computer vision algorithms (Amir et al., 2017; Maqueda et al., 2018). The second approach advocates short calculations triggered by each event, and requires a rethink of computer vision from the ground up (Benosman et al., 2012; Lagorce et al., 2015; Reverter Valeiras et al., 2016; Mueggler et al., 2017). By matching the sensor data format, this approach benefits from the sensor advantages, notably resemblance to biological signals, low latency, and data compression. Spiking neural networks fit the constraints of the second approach, and several event-based computer vision algorithms were implemented on neural simulators (Galluppi et al., 2012; Orchard et al., 2015; Haessig et al., 2018; Hopkins et al., 2018). The third approach mixes frames and events, and is well-suited to hybrid sensors, such as the *DAVIS* (Barranco et al., 2014; Moeys et al., 2016; Tedaldi et al., 2016). The framework presented in this paper is designed to encourage the second approach, though it applies to the third as well.

Given the issues arising from the Von Neumman architecture of modern computers (Indiveri and Liu, 2015), dedicated hardware seems required for event-based vision systems to match the performance of their biological counterparts. Nevertheless, microprocessors remain the de facto standard to perform general-purpose computations. They benefit from years of research and development, making them cost-effective, computationally-efficient, and user-friendly. As such, they are great tools for algorithms prototyping and early applications of event-based sensors. Furber (2017) envisions heterogeneity in future processors: general-purpose cores will work together with dedicated hardware accelerators. Under this assumption, a framework targeting CPUs is not a mere temporary solution waiting to be replaced by neural networks, but a decision support tool. It provides a baseline for algorithms power consumption and computational cost, against which implementations running on dedicated hardware can be compared. Thus, the accelerators can be chosen based on the gain they yield for tasks deemed important. A framework designed for CPUs must provide fast implementations in order to be an effective baseline. Moreover, its syntax should reflect the constrains of hardware dedicated to event-based calculations, to ease comparisons and facilitate algorithms ports from one platform to the other.

### 1.3. Frameworks

A software framework provides a collection of operators and a way to assemble them to build complex algorithms. We consider three types of frameworks related to event-based computer vision. First, we present frameworks for conventional computer vision and their limits when working with event-based data. Then, we examine event-based programming, showing how its concepts apply to event-based computer vision, even though existing frameworks were designed under constraints so different from event-based sensors that they cannot be used directly. Finally, we review frameworks dedicated to event-based computer vision.

The applications of linear algebra to a wide variety of science and engineering fields triggered, early in computer science history, the development of efficient libraries to compute matrix operations (Lawson et al., 1979). Conventional computer vision libraries use matrices to represent frames, allowing algorithms to be expressed as a sequence of operations on dense data (Thompson and Shure, 1995; Bradski, 2000; Jones et al., 2001). Dynamic, high-level languages can often be used to specify the operators order. The overhead incurred by the dynamic language is negligible when compared to the matrix operations. The latter are optimized by the underlying linear algebra library, yielding a development tool both efficient and user-friendly. Event-based computer vision is a different story. Small computations are carried out with each incoming event, and the cost of the glue between operators stops being negligible. Hence, the very structure of the libraries designed for conventional computer vision is incompatible with events, besides dealing with dense frames instead of sparse events.

Unlike event-based computer vision, event-driven programming languages and frameworks are not new: Visual Basic dates back to the 1990s. Among the concepts developed for event-driven programming, the event handler pattern and the observer pattern (Ferg, 2006) are natural choices to represent event-based algorithms and event-based cameras. Reactive programming (Bainomugisha et al., 2013), devised has a refinement over event-driven programming, introduced new abstractions to avoid state-full event-handlers and explicit time management. However, the neurons we aim at mimicking are state-full (the reaction to an input spike—for example, an output spike—depends on the current membrane potential), and fine control over time management is a needed feature for real-time systems. Hence, we choose to design our framework using event-driven rather than reactive concepts. Modern event-driven frameworks have notable applications in graphical user interfaces and web servers (Tilkov and Vinoski, 2010), where events represent user interactions and HTTP requests, respectively. The number of events per second reached in these applications is very small when compared to event-based cameras. On the one hand, a user clicking or typing does not generate much more than tens to hundreds of events per second (Cookie Clicker, 2013), and a large website, such as *Twitter* handles about six thousand requests per second on average^1^. On the other hand, an *ATIS* moving in a natural environment generates about one million events per second, with peaks reaching next to ten million events per second. The relatively small number of events the existing frameworks were designed to handle makes their design incompatible with event-based computer vision. For example, Javascript event handlers can be attached or detached at run-time, greatly improving flexibility at the cost of a small computational overhead whenever an event is dispatched.

All the frameworks dedicated to event-based computer vision circumvent the aforementioned problem using event buffers transmitted from operator to operator. The buffers typically contain a few thousand events spread over a few thousand microseconds. A typical operator loops over the buffer and applies some function on each event. The operator output consists in one or several new event buffers, looped over by subsequent operators. The sequence is dynamically defined at run-time, incurring a computational overhead. However, this cost is paid with every buffer instead of every event, becoming negligible as is the case with conventional computer vision frameworks. The first event-based computer vision framework, Jae (2007), was designed for the *DVS* and is written in Java. Subsequent cameras and their increased event throughput triggered the development of C and C++ frameworks: Cae (2007), recently re-factored and renamed DV (2019) (both from *iniVation*), *kAER*^2^ (from *Prophesee*), and *event-driven YARP* (Glover et al., 2018a,b) (developed for the *iCub*). Table 1 highlights the design differences between these frameworks. The table also includes *tarsier*, the computation component of the framework presented in this work. Unlike the other frameworks, it assembles operators at compile-time, suppressing the need for buffers between components, even though event buffers are still used to communicate with cameras or the file system.

**Table 1:** Various C/C++ frameworks provide tools to build event-based algorithms.

**Name**	**Open source**	**Operators connection**	**Dependencies**	**Communication and execution**	**Event types**
*tarsier* (this work)	Yes	Compile-time, C++ templates	–	Event-wise function calls, single thread	Template event types, contiguous memory
cAER	Yes	Run-time, XML	Boost, libpng, libusb, libuv	Event buffers, single thread	Hard-coded event types, contiguous memory
kAER	No	Run-time, C++/Python	Boost, OpenCV, Python, Qt	Event buffers, constant time intervals, single thread	Hard-coded event types, contiguous memory
Event-driven YARP	Yes	Run-time, C++/XML	Libace	IP packets, multiple programs	Hard-coded event types, contiguous memory or polymorphic event types, non-contiguous memory
Dynamic vision system	Yes	Run-time, XML	Boost, libusb, OpenCV, OpenSSL	Event buffers, multiple threads	Hard-coded event types, contiguous memory

*Despite an identical goal and programming language, they are build upon very different design decisions. Differences impact users' interaction with the framework and the performance of algorithms implementations. YARP uses IP packets, which makes it possible to run an algorithm in parallel on several machines, but adds overhead when running on a single computer*.

### 1.4. Paper Structure

This paper presents the frameworks components in the order they intervene in an actual pipeline, starting with an overall view (section 2). We introduce event-driven programming concepts and shows how they apply to event-based computer vision (section 3), followed by a brief description of *sepia*, the component implementing functions to read and write event files. Section 4 presents the design and implementation of *tarsier*, a collection of event-based algorithms. Benchmarks are used to compare its performance with existing event-based computer vision frameworks (section 5). Section 6 describes *chameleon*, a collection of Qt components to display events on a conventional screen. The implementation of drivers to communicate with event-based cameras, non-feed-forward architectures and considerations on parallelism are exposed (section 7), before discussing future work and our conclusions (section 8).

## 2. Framework Overview

**Figure 2:**
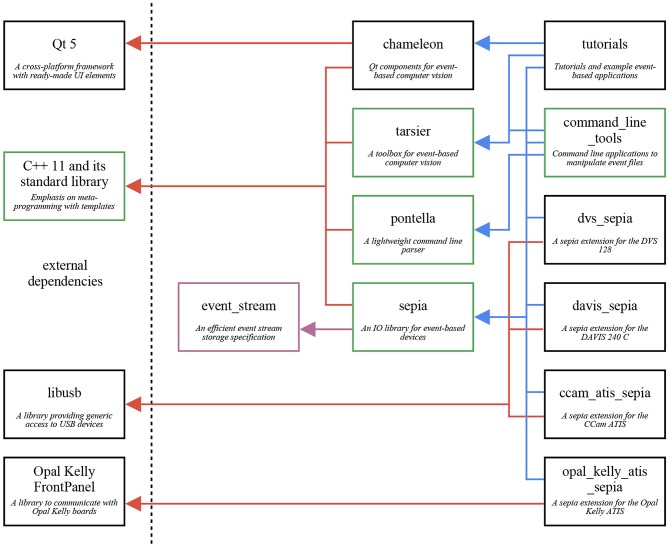
The framework presented in this paper is a collection of three independent components: *sepia* (file IO), *tarsier* (event-based algorithms), and *chameleon* (displays). Each component is hosted on its own repository, and serves a specific goal. This graph shows the three components, their external dependencies, and other repositories dependent on the framework. The *event_stream* component (purple) is not a library but a file format specification, detailed in the Appendix. The components shown in green have no external dependencies but the C++ Standard Template Library.

The framework presented in this work supports Linux, macOS, and Windows. It is organized in independent components, named after animals with unusual eyes. They work together by following the same conventions, even though they have no explicit link. This structure, illustrated in Figure 2, reduces to a minimum the external dependencies of each component, and promotes modularity. In particular, several components solely require a C++ compiler, facilitating code sharing between various machines and operating systems, and usage with other libraries. The framework's three major components are *sepia* (file I/O), *tarsier* (algorithms), and *chameleon* (display). Since these components are independent, one may use any of them without the others. For example, *sepia* can be used to read and write event files on an operating system lacking Qt support.

The framework's libraries are header-only: they require neither pre-compilation nor system-wide installation, and several versions of the library can co-exist on the same machine without interfering. Bundling dependencies with algorithms makes projects more likely to keep working over long periods of time without active support, which we believe is a critical factor for research. Moreover, an algorithm and all its dependencies can be shipped in a single zip file, making code easy to share as the supplementary material of a publication (as illustrated by this paper's Supplementary Material). Header-only libraries also simplify MSVC support for Windows (Barrett, 2014), removing the need for GCC ports, such as MinGW.

All the code is open-source, and hosted on our GitHub page (section 8). Each framework component is hosted on a distinct repository, and documented in the associated Wiki page. More importantly, the *tutorials* repository provides step-by-step tutorials and commented examples to build event-driven applications with the framework.

## 3. Event-Driven Programming

### 3.1. A Generic Event-Based Algorithm

The object-oriented *observer* pattern consist in two constructs: an observable and an event handler. The former dispatches events at arbitrary times, whereas the latter responds to each event with an action. This pattern provides a natural model for an event-based camera (observable) and an algorithm (event handler). It extends to neuron models (for example, integrate-and-fire), though implementing complex networks with feedback and delays—which can change the events order in time—is not straightforward (section 7 provides considerations on this topic). Algorithm 1 gives a generic expression of an event-based algorithm under this paradigm.

**Algorithm 1:**
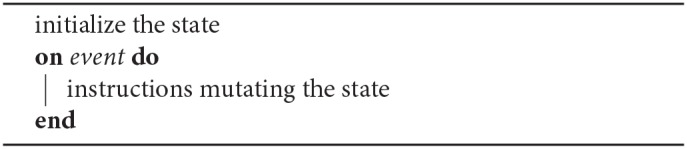
A generic event-based algorithm, or event handler.

A framework reflecting this theoretical expression facilitates algorithms implementation. A function (in the programming sense) which takes an event as sole parameter and returns nothing has a syntax close to Algorithm 1. Such a function has to mutate a state to do something useful, thus it is not a function in the mathematical sense (it is non-pure).

### 3.2. C++ Implementation

The typical C++ implementation of the observer pattern relies on dynamic polymorphism: the event handler inherits a generic class, and the observable holds a pointer to an instance of this class. This approach creates overhead for two reasons. On the one hand, every call to an event handler requires a vtable lookup and an extra dereferencing. On the other, the compiler is given less information to optimize the program.

Existing frameworks (cAER, kAER, event-driven YARP, and Dynamic Vision System) solve this issue using buffers: events are handled thousands at a time, reducing overhead proportionally. In return, user-written handlers (called *modules* in cAER, Dynamic Vision System and event-driven YARP, and *filters* in kAER) have to loop over buffers of events. Manipulating buffers, though good for performance, may foster practices that deepen the gap with neuromorphic hardware: using events ahead in the buffer to improve performance, as they are “already there,” and viewing the events as pieces of data rather than function calls. The former makes the conversion to neuromorphic hardware harder (the algorithm uses future events, increasing memory usage and latency waiting for them), while the latter strips away the event meaning (a model of a hardware spike).

**Figure 3:**
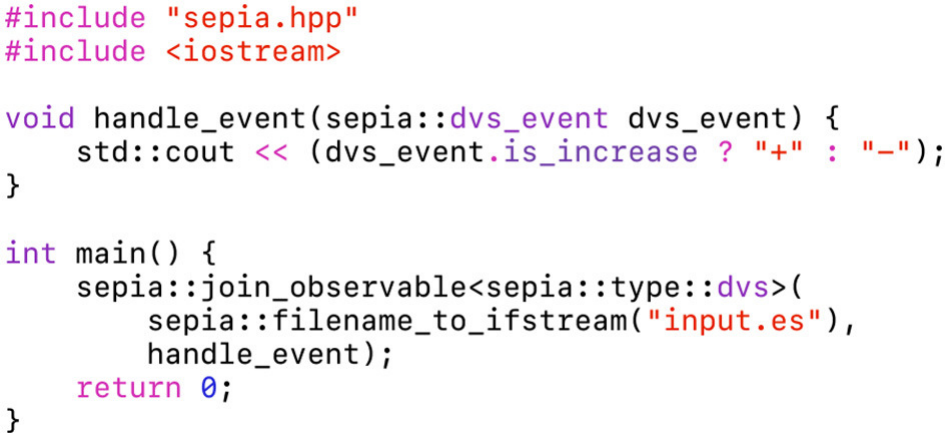
This code snippet is the “hello world” program of the *sepia* library. The function handle_event prints a plus sign in the terminal on luminance increase events, and a minus sign on luminance decrease events. The main program creates an observable from a file, with the handle_event function as event handler. This program, provided in Supplementary Material, only needs the *sepia* library in its directory to be compiled on any machine.

The presented framework relies on static polymorphism, using templates (Veldhuizen, 2000): the event handler is bound to the observable during compilation. This approach does not incur an overhead with every event, therefore buffers are not needed. The algorithm is specified by a loop-free function, illustrated in Figure 3. We want to emphasize that the code presented in this figure is a complete program, which can be compiled without prior libraries installation. The function handle_event modifies the state of the std::cout object, captured implicitly as a global variable. Events are read from the file “input.es”, which uses the *Event Stream* encoding (see the Appendix).

The *sepia* header used in this example implements file IO in the framework, and can be extended to communicate with cameras (section 7). Even though it relies on buffers, similarly to the other C++ frameworks, the event loop is hidden from the user. This is meant to reconcile two somewhat paradoxical objectives: provide a fast implementation on CPUs, which work best with bulk data, and encourage an algorithm design likely to translate to highly distributed neuromorphic systems with fine-grained calculations.

**Figure 4:**
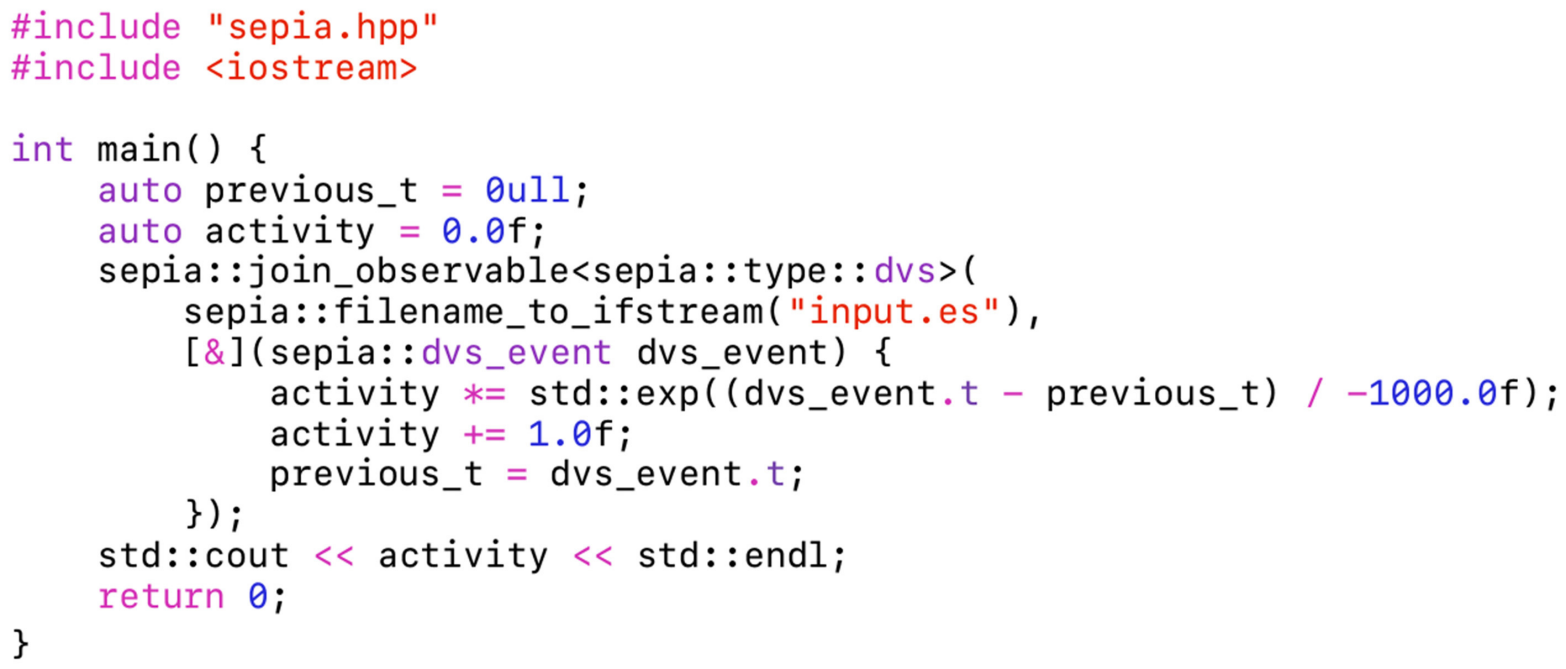
Unlike Figure 3, this program uses a lambda function to implement an event handler. Lambda functions can be declared inside the main function, keeping the global scope clean. This event handler implements a leaky neuron to compute the activity. The latter is printed once all the event from the source file “input.es” have been processed.

Static polymorphism is implemented in sepia using the same approach as the C++ Standard Template Library (see, for example, the *compare* function of the *std::sort* algorithm). Besides being efficient, it allows compile-type, type-safe “duck typing”: the code will compile as long as the syntax handle_event(event) is valid. Notably, handle_event can be a function, a lambda function or an object with an overloaded call operator. Lambda functions are great to quickly prototype an event-driven algorithm, as shown in Figure 4. This second example is a standalone, dependency-free program as well. The state variables previous_t and activity are captured by reference in the lambda function. The latter implements a sensor-wide “leaky integrate” neuron to estimate the activity, printed after processing all the input file's events.

The sepia::join_observable function blocks until all the events are processed, preventing other routines (notably Graphical User Interfaces) from running. Under the hood, it uses the GUI-compatible sepia::make_observable function, which dispatches events on another thread. In turn, this function constructs a sepia::observable object. The latter's constructor cannot be called directly, because C++ does not allow class template deduction from a constructor (until C++ 17). Thanks to the *make* function, the event handler type does not have to be explicitly specified. However, the event handler must be statically specified—not unlike connections in a neural network. Changing the event handler at run-time requires an explicit if-else block within the handler.

Both the sepia::join_observable and sepia::make_observable functions require a template parameter: the expected event type. The event handlers signature is check at compile-time, whereas the file events type is checked at run-time (each *Event Stream* file contains a single type of events).

The event handlers presented thus far have several shortcomings: they use global variables, can be used only with specific event types, and cannot be easily used from other algorithms. The *tarsier* library tackles these issues.

## 4. Building Blocks

Basic blocks that can be assembled into complex algorithms are the central feature of a framework for computer vision. They reduce development time and foster code reuse: components debugged and optimized by an individual benefit the community.

### 4.1. Partial Event Handlers

In order to represent a building block for event-based algorithms, we introduce the concept of partial event handler, illustrated by the Algorithm 2. A partial event handler is triggered by each event, similarly to the complete event handler defined in subsection 3.1. However, instead of consuming the event, the partial event handler performs a calculation, then conditionally triggers a second handler.

**Algorithm 2:**
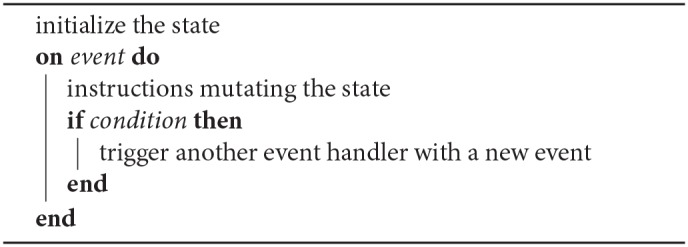
A partial event handler.

Using functions to represent handlers, we denote f_*_ a partial event handler. Since f_*_ generates events, it is an observable for a complete event handler *g*. Binding *g* to f_*_ yields the complete event handler *f*_*g*_. When called, it performs the calculations associated with f_*_, then calls *g*. Any number of partial event handlers can be chained to build an algorithm, as long as the last handler is complete. For example, with g_*_ now a partial event handler, and *h* a complete event handler, one can build the pipeline *f*_*g*_*h*__. For each child, its direct parent is an observable generating events. For each parent, its child is a complete event handler (*g*_*h*_ is a complete event handler and a child for f_*_). The syntax can be extended to partial event handlers generating multiple event types: f_*,*_ is a partial event handler with two observable types.

A more common approach to defining algorithms consists in specifying inputs and outputs for each block. However, since a partial event handler conditionally generates (possibly) multiple event types, a generic output is a list of pairs {event, boolean} representing optional objects^3^. Each boolean indicates whether the event was generated. The program assembling the pipeline would contain a complex sequence of function calls and nested if-else statements to propagate only events that were actually generated. Nested observables yield a syntax both easier to read and more closely related to the event-driven nature of the algorithm.

*f*_*g*_*h*__ is written *f* → *g* → *h* in figures to avoid nested indices. Complex pipelines, including merging and feedback, are discussed in section 7.

### 4.2. *tarsier* Implementation

The framework's *tarsier* library is a collection of partial event handlers implemented in C++. Each handler is declared and defined in a single header file: only the included ones are compiled with the program. This organization makes the code resilient to compatibility errors in unused handlers.

**Figure 5:**
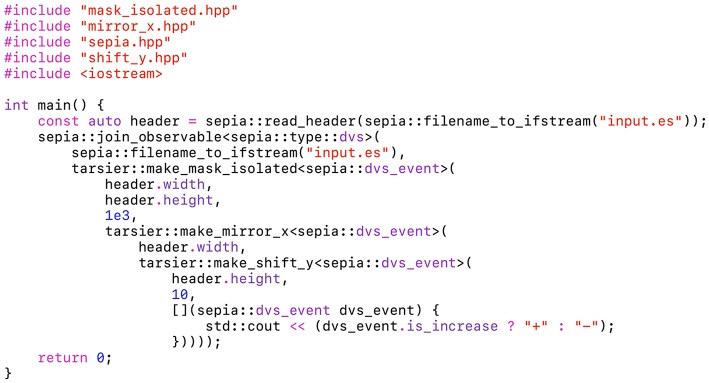
This program uses both *sepia* and *tarsier*. It can be compiled on any computer without installing external libraries. The pipeline is implemented as a sequence of nested partial event handlers. tarsier::mask_isolated removes noisy events, tarsier::mirror_x inverts the *x* coordinate and tarsier::shift_y shifts the *y* coordinate by a fixed offset. Events outside the original window after shifting are not propagated.

The partial handlers are implemented as classes with an overloaded call operator. The children handlers types are templated. In order to allow type deduction, each class is associated with a *make* function: the partial event handler f_*_ is associated with *make*_*f*. For any complete event handler *g*, *make*_*f*(*g*): = *f*_*g*_. Pipelines are built by nesting *make* functions: *make*_*f*(*make*_*g*(*h*)) = *f*_*g*_*h*__. Unlike event handlers, the high-order *make* functions are pure. Most of them take extra parameters to customize partial event handlers. For example, tarsier::make_mask_isolated, which builds a partial event handler propagating only events with spatio-temporal neighbors, takes a sensor width and height and a time window as parameters. Figure 5 shows a simple *tarsier* pipeline, bound to a *sepia* observable.

The *tarsier* and *sepia* libraries are compatible even though they are not explicitly related. Every partial event handler provided by *tarsier* uses template event types, besides template event handlers parameters. The event type has to be specified explicitly (sepia::dvs_event in Figure 5), and must have a minimal set of public members which depends on the event handler (often *x*, *y* and *t*). A C++ struct with at least these three fields meets the requirements of most *tarsier* handlers. Users can define custom types to best represent the events output by their algorithms (flow events, activity events, line events, time surfaces…), or to customize the events payload (with a camera index for stereo-vision, sparse-coding labels…).

**Figure 6:**
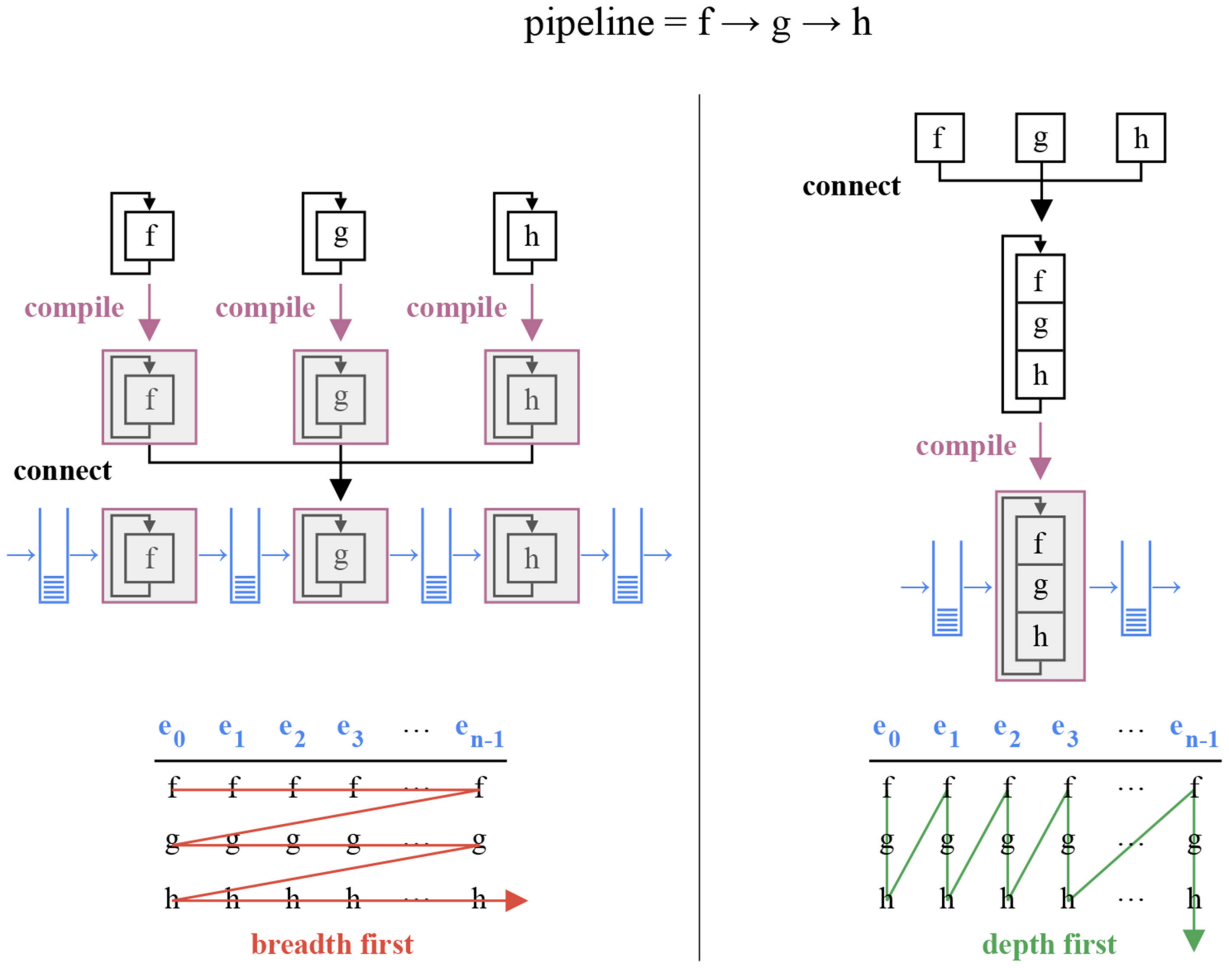
The 3 × *n* operations associated with a sequence of three event handlers *f*, *g*, and *h* and a buffer of *n* events *e*_*i*_, *i* ∈ [0…*n* − 1] can be performed in two orders: breadth first and depth first. An implementation relying on dynamic polymorphic incurs an overhead for every distinct function call, and must therefore use the breadth first approach (left). Depth first yields lower latencies, but requires static polymorphism: the pipeline must be assembled during compilation (right).

This implementation has several benefits. Since the pipeline is assembled statically, type checks are performed by the compiler. Missing event fields and incompatible observable/event handler bindings are detected during compilation, and meaningful errors are returned (in contrast with run-time segfaults). Moreover, an event loaded from disk or sent by a camera, with a specific type, can be used directly without an extra copy to a buffer holding events with another type. Since the compiler manipulates a completely specified pipeline, it can perform more powerful code optimizations. Finally, since static event handler calls have no run-time overhead, events buffers can be traversed depth-first instead of breadth-first (Figure 6). This operation ordering reduces the pipeline latency, as observed in section 5.

## 5. Comparative Benchmarks

Event-based computer vision shows promise for real-time sensing on robots (Blum et al., 2017). If a CPU is used to run computer vision algorithms on a robot, the code efficiency can make the difference between a real time and non-real time system. Performance is also essential to make realistic comparisons of conventional hardware and neuromorphic hardware, or to compare two event-based CPU algorithms. Even though the average number of operations per event gives an estimation of an algorithm complexity, it does not account for compiler optimizations, memory IO or processor optimizations (branch predicting, cache…). Hence, accurate speed comparisons require a comparison of implementations, whose result depends on the quality of the implementations.

The efficiency of an implementation depends on many parameters, including the algorithm itself, the choice of programming language, the use of suitable programming primitives, and the properties of the framework. We aim to compare the contribution of the latter among frameworks designed for event-based computer vision. We restrict this comparison to frameworks written in C/C++, to avoid comparing languages rather than frameworks. The compared algorithms are given the same implementation in each framework, thus observed differences can only be attributed to frameworks properties.

The present benchmarks focus on event processing: the *tarsier* library is compared to its counterparts in *cAER, kAER*, and *event-driven YARP*. The other frameworks components (file IO, camera drivers and display) are not considered. Moreover, we were not able to include *Dynamic Vision Systems* in the benchmarks: its current implementation uses multiple threads and circular FIFOs between modules. Modules running faster than their children overflow the FIFO, resulting in silent event loss. Though not critical for real-time applications, this loss biases benchmark results and prevents graceful program termination, which depends on exact event counting. Nevertheless, since the structural design choices of *Dynamic Vision Systems* are similar to those of *cAER*, we expect comparable results. *Event-driven YARP* offers two implementations for event buffers: vectors and *vQueues*. Vectors leverage contiguous memory, whereas *vQueues*, which are double-ended queues of pointers to polymorphic events, support arbitrary types. We evaluate the performance of both options. The results associated with the vector (respectively *vQueue*) implementation are labeled *YARP* (respectively *YARP vQueue*). The code used to run the benchmarks is available online (section 8). This resource also illustrates the implementation of the same algorithms in various frameworks.

Before each benchmark, we load a specific stream of events in memory. The events are organized in packets of up to 5, 000 events and up to 10 ms (a new packet is created as soon as either condition is met), as to mimic the typical output of a camera. We consider two performance indicators. The *duration* experiment measures the total time it takes to read the packets from memory, run an algorithm and write the result back to memory. It indicates how complex a real-time algorithm can be. The *latency* experiment measures the time elapsed between the moment a packet is available and the moment results are written to memory. A packet is made available when the wall clock time goes past the timestamp of its last event. A busy-wait loop is used to wait for the wall clock time if the framework is ready to handle a packet before the latter is available. This mechanism simulates the output of an actual event-based camera while avoiding putting processes to sleep, which is a source of non-deterministic variations in the measured latency. The packets contain sepia::dvs_event objects, chosen as a neutral type for all the frameworks. Event type conversions, if needed, are taken into account in the performance measurement. This choice is not an unfair advantage to *tarsier*, since its handlers are compatible with any event type (including the types provided by *sepia*). The events dispatched from one partial event handler to the next are framework-dependent. However, to avoid uneven memory writes, the output events are converted to a common type before being pushed to a pre-allocated vector. To make sure that the output is not skipped by the compiler as an optimization, we calculate the MurmurHash3 (Appleby, 2014) of each output field once the algorithm completed. The resulting values are controlled for each benchmark run, and guarantee that each implementation calculates the same thing.

**Figure 7:**
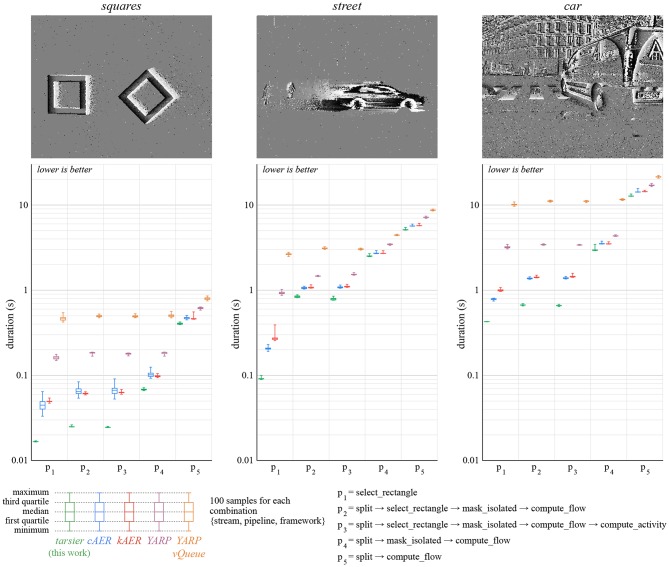
We implement the same partial event handlers in each framework in order to compare them. We consider five pipelines and three event streams. The total time it takes to handle every event from the input stream is measured 100 times for each condition. We attribute the better performance of *tarsier* to static polymorphism, which yields a program with fewer memory operations.

**Figure 8:**
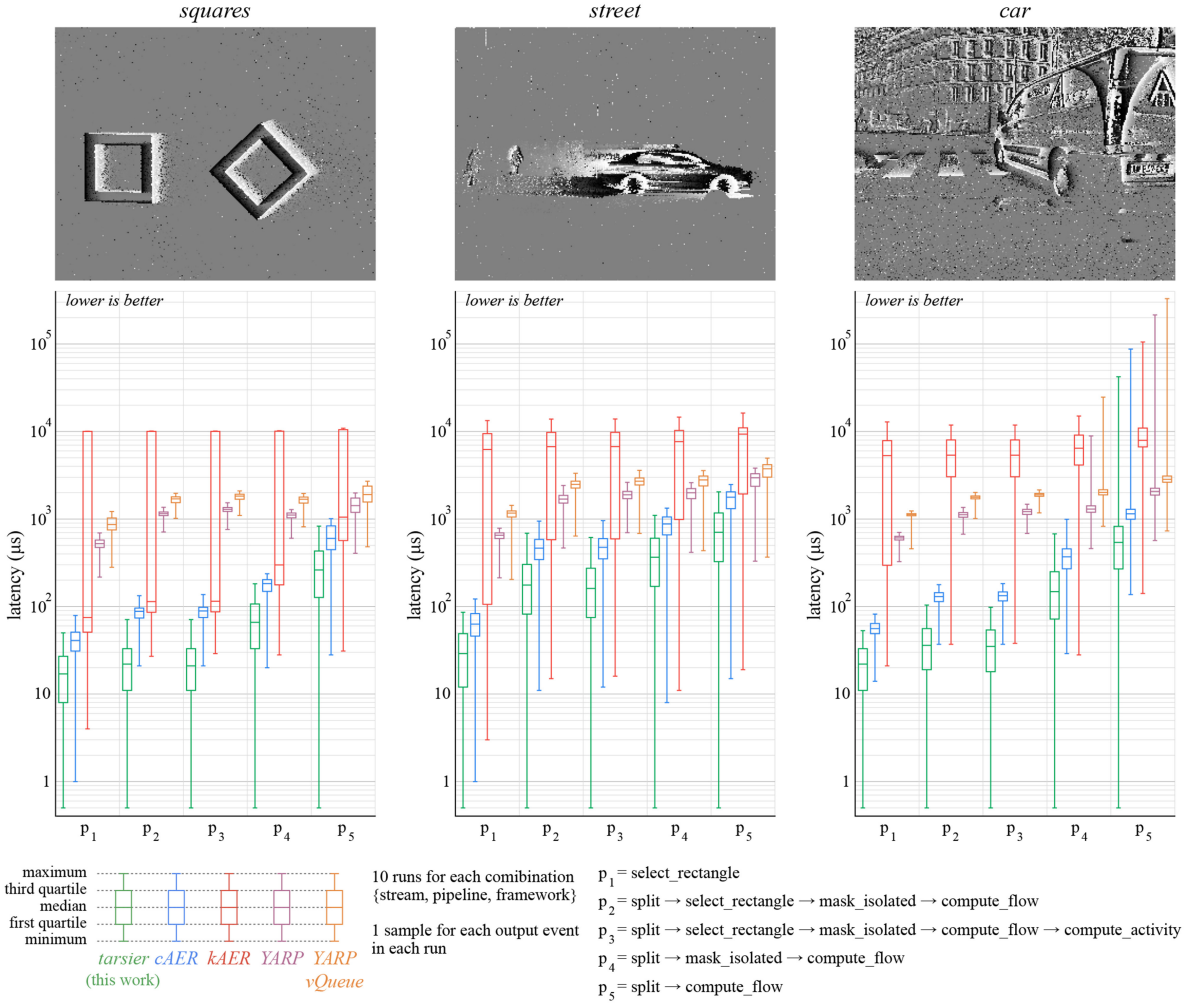
Low-latency is an important feature of event-based cameras, and therefore event-based frameworks. We measure the time elapsed between the moment a buffer is available and the moment associated output events are produced by the pipeline. Events that are not propagated by the pipeline (for example, removed noise) are not taken into account. For each condition, latency is measured for each output event over 10 runs of the whole stream. We attribute the better performance of *tarsier* to depth-first traversal. *kAER* under-performs in this benchmark since it constrains buffers duration, unlike the camera model assumed in the benchmarks: the resulting buffer reorganization increases delays. This benchmark's relative variations are larger than the *duration* benchmark's variations. The same time measurement functions are used, however durations are order of magnitude larger than latencies.

The benchmarks use five distinct algorithms (*p*_1_ to *p*_5_) described (Figures 7, 8). Each pipeline is assembled from one or several of the following partial event handlers:

- select_rectangle only propagates events within a centered 100 × 100 pixels window.
- split only propagates events representing a luminance increase.
- mask_isolated only propagates event with spatio-temporal neighbors.
- compute_flow calculates the optical flow.
- compute_activity calculate the pixel-wise activity. The activity decays exponentially over time, and increases with each event.

We use three event streams, listed in Table 2 and available in the benchmarks' repository. These streams contain polarity events recorded by an ATIS, in both controlled and natural environments. The *duration* experiment is run one hundred times for each combination {stream, pipeline, framework}, and the *delay* experiment ten times. Each *delay* task generates many samples, whereas each *duration* task yields a single value. All 6,600 tasks are shuffled, to avoid possible biases, and run sequentially on a computer running Ubuntu 16.04 LTS with an Intel Core i7-6700 CPU @ 3.40GHz CPU and a 16 GB Hynix/Hyundai DDR4 RAM @ 2.4 GHz. The code is compiled with GCC 5.5, C++11 and the -O3 optimization level.

**Table 2:** We use three event streams recorded by an ATIS to perform benchmarks.

**Stream name**	**Description**	**Duration (s)**	**Event rate (s^**−1**^)**
Squares	Artificial scene, moving geometric shapes, fixed sensor	9.50	2.83e5
Street	Natural scene, moving pedestrians and cars, fixed sensor	50.6	3.17e5
Car	Natural scene, sensor inside a moving car	69.6	9.56e5

*The streams were chosen for their different conditions (artificial and natural scenes, fixed and moving sensor) and average event rates*.

### 5.1. Duration

The *duration* benchmark results are illustrated in Figure 7. The approach presented in this paper yields the smallest duration on all the pipelines and event streams considered. This improvement over state-of-the-art frameworks can notably be attributed to a reduced number of memory reads and writes, thanks to the template event types.

*Event-driven YARP* yields longer durations than the other frameworks. The difference is most likely related to the use of IP packets to communicate between filters. The alternative implementation *event-driven YARP vQueue* is substantially worse with respect to the considered benchmarks. We attribute the performance loss to the non-contiguous memory allocation of events in *vQueues*. The other frameworks use either multiple hard-coded event types (*cAER, kAER, event-driven YARP*), or template event types (*tarsier*) to leverage contiguous memory.

The pipeline *p*_3_ contains more operations than *p*_2_. Yet, the *p*_3_ *tarsier* implementations has a smaller duration than *p*_2_ (the effect is most visible with the *street* stream). The compute_activity event handler does not utilize the visual speed calculated by compute_flow, only the flow events' timestamp and position. Therefore, the flow computation can be skipped without changing the algorithm outcome. In the case of frameworks with modules assembled at run-time, the compiler cannot make this simplifying assumption. We believe this behavior can improve the performance of complex pipelines, where finding redundant or unused calculations manually can prove difficult.

### 5.2. Latency

The *latency* benchmark results are illustrated (Figure 8). Wall clock time is measured with microsecond precision for each input packet and each output event. Latency samples are calculated by subtracting the wall clock time of output events and that of their input packet. In some cases, the latency is zero, meaning that the actual elapsed wall clock time is smaller than the measurements' precision. To allow representation on a log-scale, we round up null latency samples to 0.5 μs.

The relative standard deviation is much higher for the *latency* benchmark than the *duration* one. As a matter of fact, measured values are much smaller: durations are in the order of seconds, whereas latencies are on the order of microseconds. Thus, every small non-time-deterministic operation (memory operations, CPU prediction, kernel preemption…) has, relatively, more impact.

The *kAER* framework yields substantially larger latencies than the other frameworks. Since it enforces buffers with a constant duration, latency increases when the buffers provided by the camera use a different, possibly variable, duration.

**Figure 9:**
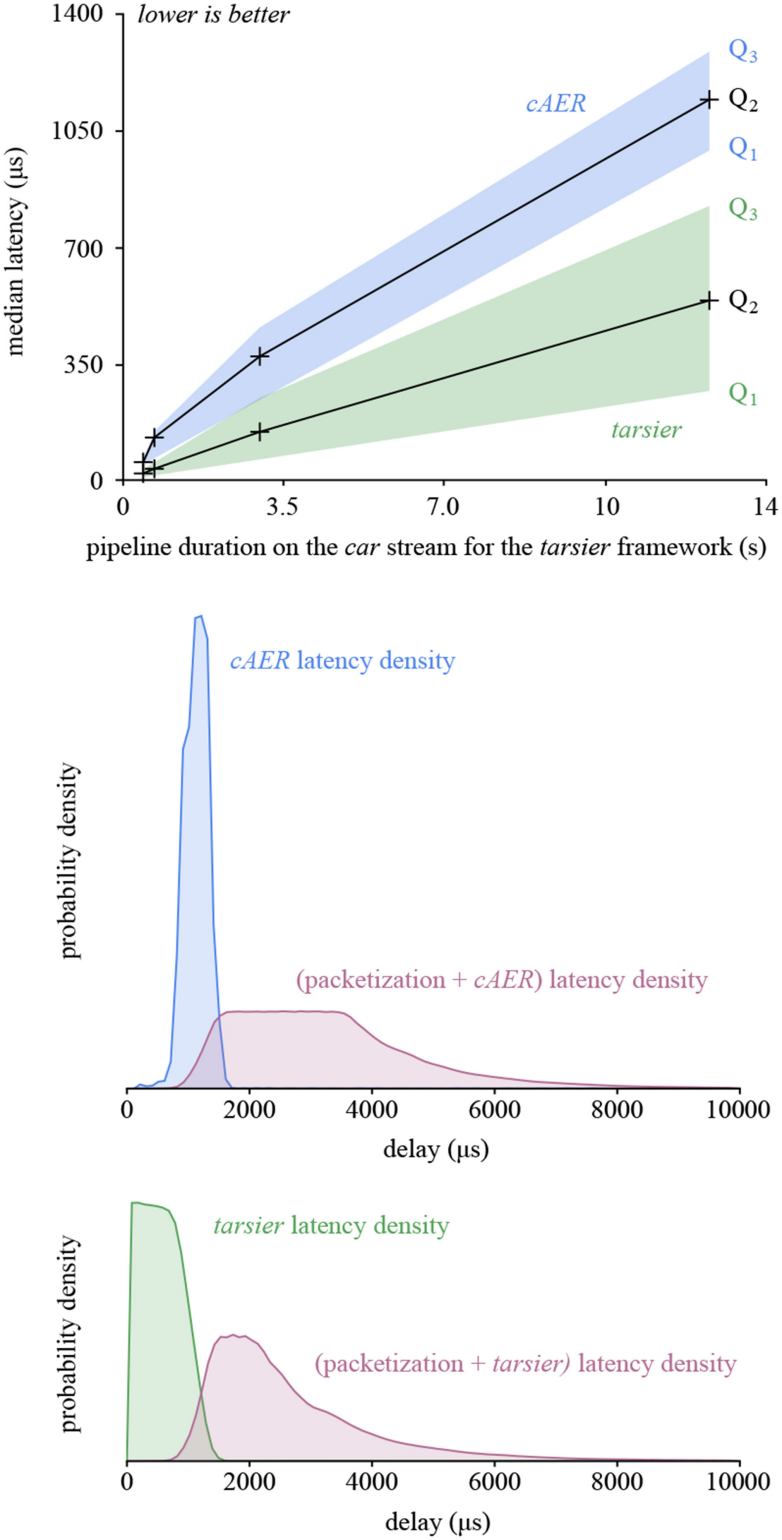
The graphs presented in this figure take a closer look at the latency created by *tarsier* and *cAER* for the *car* stream. In the top graph, latency is plotted as a function of pipeline duration when run with *tarsier* (arbitrarily chosen as a complexity indicator). *tarsier* has a smaller median density, but a larger variance. The density probability for the most complex pipeline is plotted in the middle and bottom graphs (blue and green). It accounts only for framework latency (as does the first graph). Adding the latency caused by packetization in the camera (before the USB transfer) yields the total latency. The depth-first traversal leveraged by *tarsier* better counterbalances packetization, resulting in both a lower total latency and a smaller variance.

The framework presented in this paper outperforms the others in this benchmark as well. Low-latency can be a major benefit for robots or closed-loop systems. The performance gain is a consequence of buffer depth-first traversal and the reduced number of memory operations, since inter-handler communication is not implemented with buffers. The latency reduction improves with the duration of the algorithm when comparing *tarsier* and *cAER*, as illustrated in Figure 9 (top graph).

However, the latency variance is larger for *tarsier* than *cAER*, and increases with the pipeline duration as well. This is another consequence of depth-first traversal: the first event in the input buffer is handled as soon as the packet is available, and therefore has a small latency. In contrast, the last event in the buffer waits for all the other events to be handled, resulting in a much larger latency. This phenomenon does not exist with *cAER* since the whole packet is processed by each module sequentially: events with the same input packet exit the pipeline at the same time.

The latency used so far takes only the framework into account. The first event of each buffer is also the one that waited the most in the camera while the input buffer was being filled. If we neglect the USB transfer duration, we can define the total latency associated with an event as the sum of the framework latency and the timestamp difference between the last event in the packet and the considered event. The total latency as well as its variance are both smaller for *tarsier* when compared with *cAER*, since the packetization effect is counterbalanced by the depth-first traversal. Both the framework latency and total latency densities are illustrated in Figure 9 (bottom graphs).

## 6. Event Displays

**Figure 10:**
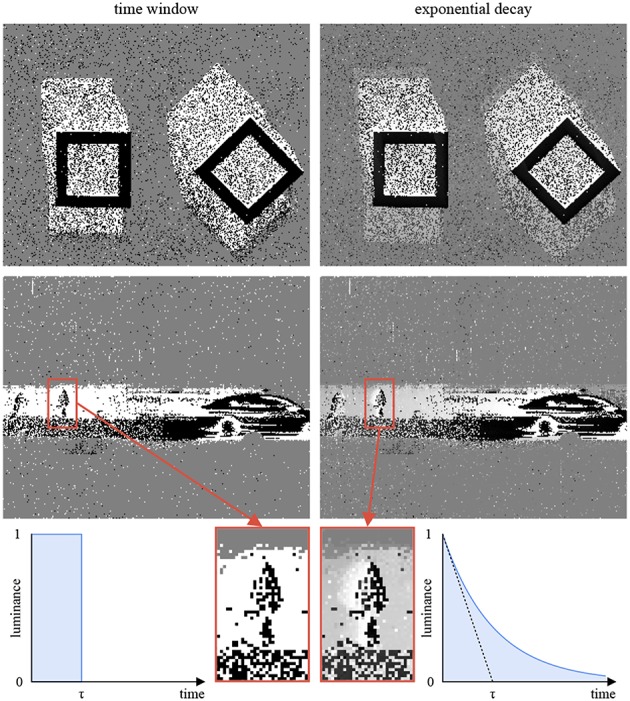
This figures compares two strategies to convert events to frames for display. The time window approach (left) degrades temporal information: the still frames do not hold enough information to determine the geometric shapes motions (top row) or the relative speed of the car and the pedestrian (bottom row). The exponential decay approach (right) represents temporal information with gray levels. It is computationally more expensive than the time window approach, but can be easily implemented on a GPU to relieve the CPU.

Conventional screens display frames at fixed time intervals^4^. In order to display events, one has to perform a conversion. Most frameworks rely on fixed time windows: a frame pixel is colored in white if it was the source of a luminance increase event during the associated time interval, in black if the luminance decreased, and in gray if nothing happened. This approach does not account for the high temporal resolution of the signal. Another method relies on time decays (Cohen, 2016; Lagorce et al., 2017): the frame pixel *i* is given the color $c_{i}=\frac{1}{2}\left(1+\delta_{i}\cdot exp\left(-\frac{t-t_{i}}{\tau }\right)\right)$. *t* is the current timestamp. *t*_*i*_ is the timestamp of the most recent event generated by the pixel *i*. δ_*i*_ = 1 if the last event generated by *i* corresponds to a luminance increase, and −1 otherwise. τ is a fixed decay. Figure 10 illustrates the difference between the two methods, highlighting the benefits of exponential decays.

The full-frame decay rule requires an exponential calculation on every event for every pixel (for an ATIS, 72,960 pixels a million times per second), which is both unrealistic and unnecessary, since the typical display features a 50 Hz refresh rate. Instead, one can calculate the decays only when a frame is about to be rendered, and use the GPU available on most machines to do so. GPUs are designed to run massively parallel calculations with every frame, thus are well-suited to this task.

**Figure 11:**
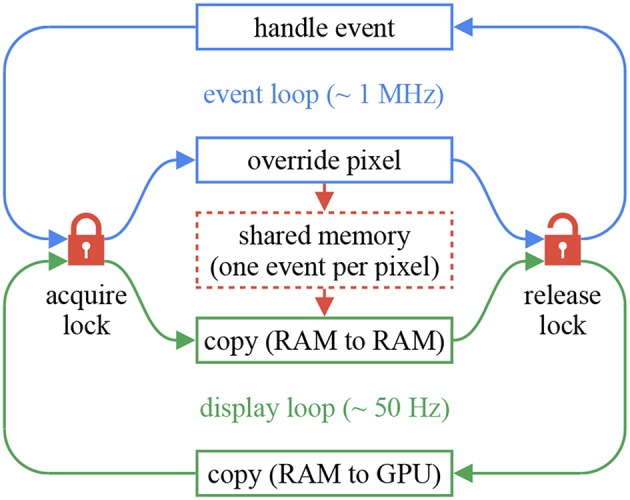
In order to convert events to frames, one has to reconcile the very different rates of the event loop (about 1 MHz) and the display loop (often 50 Hz). We use a shared memory the size of the sensor, protected by thread-safe locks. On each event, the first thread (blue) overwrites former events with the same spatial coordinates. Every time a frame is about to be rendered, the display loop (green) copies the shared memory to RAM and releases the lock, then communicates with the GPU. The memory-to-memory copy minimizes lock ownership, to avoid blocking the event loop. The lock, acquired with every event, is implemented as a spin-lock mutex.

**Figure 12:**
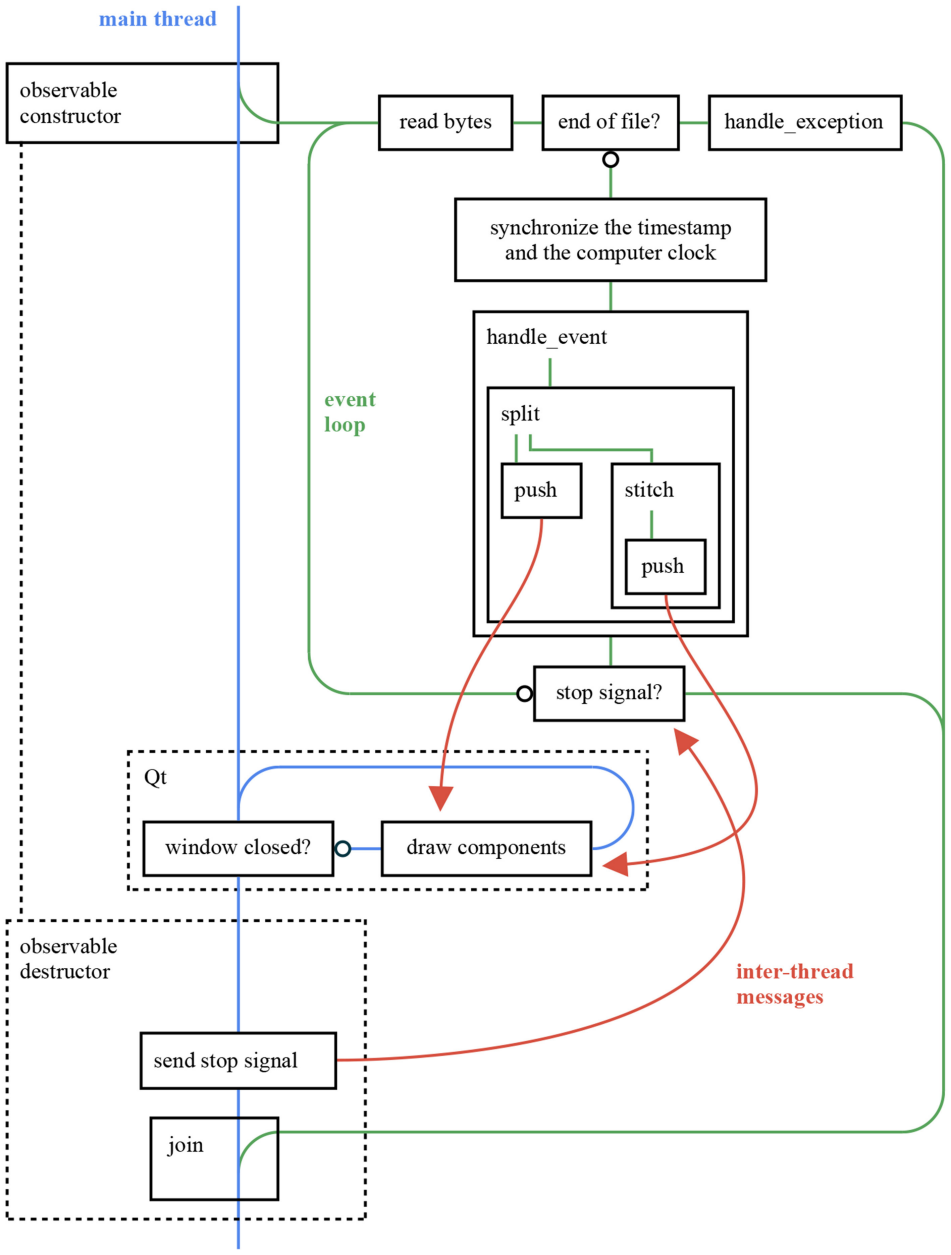
This figure provides an overall view of the threads in an ATIS event stream viewer application using *sepia, tarsier*, and *chameleon*. The application reads an *Event Stream* file and displays it as frames. The observable constructor (respectively destructor) creates (respectively joins) the event loop thread, in accordance with the RAII (Resource acquisition is initialization) philosophy of C++. The *push* inter-thread messages rely on the mechanism illustrated in Figure 11, whereas the *stop* signal is implemented as an atomic boolean. The code for this application can be found in the *tutorials* repository.

The *chameleon* library provides Qt (1995) components to build event displays. The components are independent and header-only. Unlike *sepia* and *tarsier, chameleon* cannot be used without *Qt 5*. In return, the event displays can easily be integrated into complex user interfaces. The chameleon::dvs_display implements the full-frame decay method mentioned previously. This component assumes two threads: an event loop (for example, a *sepia* observable followed by a *tarsier* pipeline) and a render loop. The loops communicate using a shared memory with one cell per pixel, where the last timestamp and polarity of each event is stored. When a new frame is about to be rendered, the shared memory is sent to an OpenGL program to compute each pixel's time decay. The shared memory is accessed millions of times per second by the event loop. Usual mutexes can cause non-negligible overhead, since they rely on system calls. The *chameleon* implementation uses spin-lock mutexes instead (essentially busy-wait loops with atomic variables), at the cost of increased CPU usage. To minimize the strain on the event loop, the render loop first creates a local copy of the shared memory, then releases the mutex, and finally communicates with the GPU. This mechanism is illustrated in Figure 11. Figure 12 gives an overview of an application build with the three major components of the framework, with a focus on thread management. This application's code is available in the *tutorials* repository.

The proposed approach does not rely on pre-defined frame boundaries: the frame-rate matches the display rate regardless the event loop speed. Consequently, the visual animation remains smooth even if the event pipeline is slower than real time. A smooth slow-motion display can be created by artificially slowing down the event loop.

The colors used by the DVS display can be customized: the *c*_*i*_ value is then used as a weight parameter for mixing the colors. Transparent colors can be used, enabling display overlays for cameras generating multiple stream types (such as the ATIS or the DAVIS). Other notable components provided by *chameleon* include a vector field display (well-suited to flow events), a blob display, a time delta display (to represent the absolute exposure measurements of an ATIS), and a screen-shot component to easily create frame-based videos. These components use template event types, similarly to *tarsier* event handlers, and the type requirements follow the same conventions. The displays coordinates system follows the usual mathematical convention, with the origin located at the screen's lower-left pixel. The usual computer vision convention (origin at the upper-left pixel) is not used as it is a result of the matrix representation of frames, which event-based algorithms aim to avoid.

## 7. Framework Extensions

### 7.1. Camera Drivers

Since most event-based cameras feature a USB interface, their drivers can be devised as user-space programs atop a third-party library overseeing the USB communication. To keep the codebase modular and minimize dependencies, each camera interface is held in a distinct repository extending the *sepia* library.

As of now, the following cameras are supported:

- DVS 128. We re-implemented the *libcaer* interface to provide out-of-the-box MSVC support.
- ATIS (Opal Kelly board). This extension depends on the non-free Opal Kelly Front Panel library.
- ATIS (CCam 3). This camera has the same pixels and arbiter as the Opal Kelly ATIS, however it features a custom FPGA and a USB 3 interface. It was designed by Prophesee.
- DAVIS 240C. We re-implemented the *libcaer* interface for this sensor as well.

**Figure 13:**
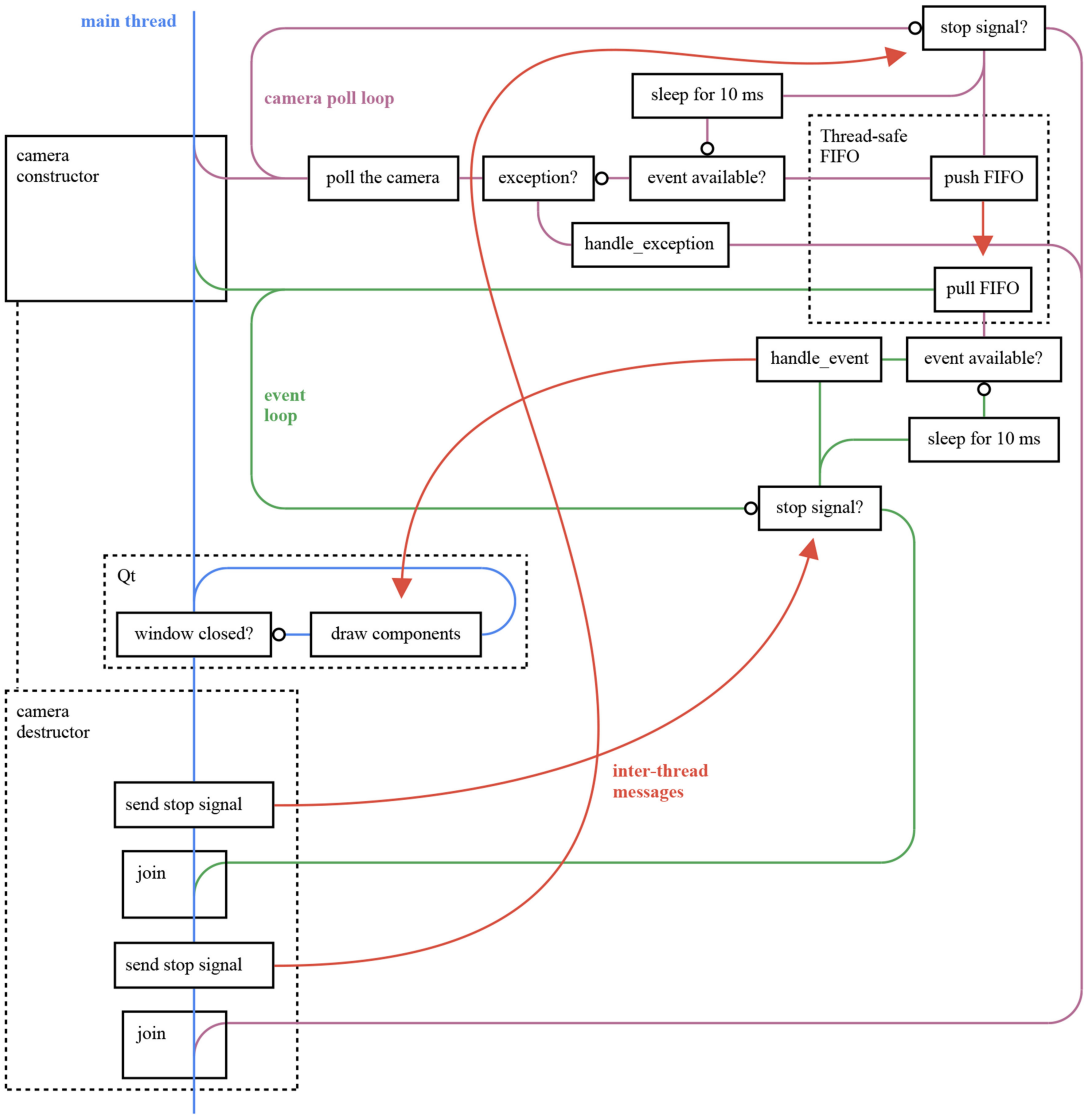
This figure provides an overall view of the threads in an ATIS camera viewer application using *opal_kelly_atis_sepia, tarsier*, and *chameleon*. The application listens to a camera and displays the generated events as frames. It encompasses the application illustrated in Figure 12. The extra thread is used to communicate with the camera as fast as possible even when the event loop is busy. The two threads communicate through a thread-safe FIFO buffer implemented in *sepia*. The Opal Kelly Front Panel library does not provide a *poll* function, hence the explicit *sleep* step in the graph. However, this function is used by *sepia* extensions based on libusb, resulting in reduced CPU usage.

Event-based cameras have internal buffers to store events while waiting for a USB transfer. A camera generating events at a faster rate than what the computer program can handle ends up filling its internal buffers to capacity. At this points, cameras either drop new events or shuts down. To circumvent this issue, each *sepia* extension uses an extra thread to communicate with the camera, independently of the event loop executing the algorithm. The two threads communicate with a thread-safe circular FIFO. An overall view of the threads of an application using a *sepia* extension, *tarsier* and *chameleon* is given in Figure 13. The circular FIFO implementation is provided by *sepia*.

Multiple parameters can be specified to configure an event-based camera, such as the operating mode or the current biases. JSON files are used by *sepia* extensions to specify the configuration. The *sepia* header implements a JSON parser and validator to load configuration files and warn users in case of syntax errors or unknown parameters.

### 7.2. Complex Pipelines

The present framework is designed to implement feed-forward pipelines, with optional splits. Most partial event handlers can be represented with populations of neurons, as they perform small calculations with each input. Thus, event-based pipelines can be translated to neuromorphic hardware, though a method to actually perform the conversion has yet to be devised.

However, not all neural networks can be represented with event handlers. Notably, neurons with second order dynamics and synapses with delays dispatch events that are not an immediate response to an input spike. The present framework, and more generally, purely event-based algorithms—cannot implement such models. To use complex neurons to process the output of a camera, one needs to leverage frameworks designed to implement neural networks. The present framework can, in this case, be used to communicate with sensors, perform low-level processing and send events to the neural network.

Nevertheless, two types of architectures more complex than feed-forward pipelines can be implemented in our framework: streams merging and feedback loops. Even though they still impose more constraints than generic spiking neural networks, they allow for the efficient implementation of algorithms on a CPU without the need for another framework.

Streams merging has the following generic structure:


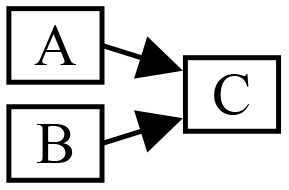


**A**, **B** are partial event handlers, and **C** is a complete event handler. This structure appears when merging the results of several calculations with a common origin. For example, one may split a stream of polarity events to compute two optical flows (one per polarity) and merge them to calculate an overall flow. **A** and **B** run sequentially in this scenario, therefore events are dispatched to **C** in the order of their timestamps. This scenario can be implemented by constructing **C** before the pipeline. The partial event handlers **A** and **B** are both given a reference to **C** as complete event handler. The std::reference_wrapper class can be used to prevent template deduction to a non-reference type, which would trigger a copy.

The merge operation can also arise from the use of multiple sensors, for example for stereo-vision or audio-video fusion. In this case **A** and **B** run in parallel, on different threads. Given the non-deterministic scheduling of most operating systems, **C** must re-order the events dispatched by its observables before handling them. This operation is implemented by the partial event handler tarsier::merge, compatible with an arbitrary number of observables.

A simple feedback loop can be modeled as:


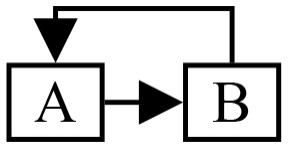


**A** and **B** are both partial event handlers. This structure can be useful for flow control or learning. The feedback operation can be executed at various moments in the lifecycle of an algorithm: after processing a batch of data, immediately after each event and after each event with a delay. The implementation of the second and third approaches is not straightforward with existing packet-based frameworks. The whole packet has already been processed by **A** when the first event is processed by **B**, preventing the associated feedback from affecting the next events. The second approach can be implemented in *tarsier* using variables shared between **A** and **B**. Before handling an event, **A** reads from the variables and processes the event accordingly. After handling an event, **B** writes to the variables. Since an event is completely handled before the next is considered, modifications of the shared variables caused by the event *n* will be available to event *n*+1. The third approach—adding delays to the feedback—can be implemented by combining the second approach and a merge structure.

### 7.3. Parallelism

The application illustrated in Figure 13 relies on multiple threads, and can take advantage of CPUs with a few cores. However, the sequential strategy presented so far does not harness the full potential of many-cores architectures.

The creation of parallel tasks and inter-task communication have a cost. An application using multiple tasks must reach a compromise on grain size (Acar et al., 2018). A large grain size yields less overhead, whereas a small grain size fully utilizes the CPU capabilities. The atomic tasks of an event-based pipeline are its partial event handlers. Larger grain sizes can be obtained by combining several partial handlers into a single task. The tasks represented in Figure 6 can be combined either vertically (one thread per event) or horizontally. The former requires inter-thread communication with every partial handler to ensure sequentiality, canceling the benefits of parallelism, whereas the latter corresponds to the buffer-based approach of *event-driven YARP* and *Dynamic Vision System*. Consequently, latency increases with the grain size.

Parallelism can be beneficial when high latency is not critical and a high throughput is required. However, implementing parallelism efficiently is not straightforward: to avoid FIFO overflows between modules, possibly complex flow control algorithms must be implemented. High-quality libraries provide high-level tools to build parallel algorithms, such as Intel Threading Building Block's flow graph (Tovinkere and Voss, 2014). The partial event handlers provided by *tarsier* can be integrated with such tools. Thus, one can implement an algorithm once and use it with either a low-latency *tarsier* pipeline or a high-throughput flow graph. An example integration of a partial event handler in a class manipulating buffers is given in the *tutorials* repository. This approach can also help integrating *tarsier* with other event-based frameworks, in order to use existing drivers and viewers.

## 8. Conclusion and Discussion

We have presented a modular framework for event-based computer vision with three major components: *sepia, tarsier*, and *chameleon*. The components, though designed to work together, have no explicit relationship, thus minimizing the external dependencies of each component. Moreover, each component can easily be replaced with other libraries.

The presented framework hides buffers from the user, serving our goal: encouraging functional, event-based semantics likely to translate to neuromorphic hardware while providing an efficient implementation on CPUs. Benchmarks show an increased throughput and a reduced latency compared to state-of-the-art frameworks for event-based computer vision. Using contiguous memory to store events is crucial to performance. Moreover, assembling pipelines before compilation reduces latency and improves throughput, thanks to better compiler optimizations and fewer memory operations. The common practice of hard-coding simple operations (mirroring the stream, removing noise…) in file readers to reduce latency is no longer required with static polymorphism, yielding a cleaner, more generic codebase.

The benchmarks compare performance with pipelines of varying complexity. However, all the considered experiments use simple pipelines (without merges or loops), focus solely on the algorithm performance (the performance of IO and display operations is not evaluated), and run in real-time on the test machine. In a future work, we plan to devise new benchmarks to cover more use-cases. Moreover, adding more measurements, such as power consumption, will enable comparisons with neuromorphic hardware.

Assembling a pipeline before compiling requires meta-programming, i.e., another programming language to generate the actual code. The framework presented in this work uses C++ template meta-programming, since this language is supported by every standard-compliant compiler. Nevertheless, it can be unsettling to new users, and makes the creation of wrappers in high-level languages, such as Python, difficult. A high-level language or graphical user interface must bundle a C++ compiler to generate *tarsier* pipelines. Nevertheless, the framework modular structure and its independence from third-party libraries make it a good candidate for a common low-level library to multiple high-level interfaces. It can notably be integrated with native Android applications, or used to speed up Python modules.

The observer pattern used by the framework naturally models event-based cameras and algorithms. However, this pattern can lead to the problem known as callback hell: deeply nested statements make the code hard to read. Languages, such as Javascript have solved this problem with the async/await construct. This construct is available in C++, but is not compatible with the template deduction mechanism leveraged by the framework.

The current implementation of *partial event handlers* relies on *make* functions. These functions wrap the handlers constructors to enable template deduction. The C++17 standard allows template deduction from the constructor of a class, making the *make* functions unnecessary. The upcoming Debian 10 and macOS 10.15 operating systems will provide full support for this standard with their default libraries, allowing a major framework update.

## Data Availability Statement

The code repositories mentioned in this study:

- framework tutorials https://github.com/neuromorphic-paris/tutorials
- frameworks benchmarks https://github.com/neuromorphic-paris/frameworks_benchmarks
- *sepia* repository https://github.com/neuromorphic-paris/sepia
- *tarsier* repository https://github.com/neuromorphic-paris/tarsier
- *chameleon* repository https://github.com/neuromorphic-paris/chameleon
- *Event Stream* specification https://github.com/neuromorphic-paris/event_stream.

## Author Contributions

AM, S-HI, and RB contributed the conception and design of the study. AM devised the theoretical model, implemented it, carried out the experiments, analyzed the results, and wrote the first draft of the manuscript. All authors contributed to the manuscript revision, read, and approved the submitted version.

### Conflict of Interest

The authors declare that the research was conducted in the absence of any commercial or financial relationships that could be construed as a potential conflict of interest.

## Footnotes

^1^*CBS News Uses Twitter as Part of Its News Investigating and Reporting* (2015). Available online at: https://developer.twitter.com/en/case-studies/cbs-news

^2^*kAER 0.6*, used in this work, is the latest version developed by our laboratory and licensed to *Prophesee*. Newer versions are now developed and maintained by *Prophesee* and the source codes are for internal use only, hence their performances are not assessed in this work.

^3^Using C++ STL primitives, the output's type would be std::tuple<std::pair<event_type_0, bool>, std::pair<event_type_1, bool>, …>. With the C++ 17 standard, std::optional<event_type> can be used instead of pairs.

^4^Recent screens compatible with Nvidia's G-Sync technology can display frames at varying time intervals, narrowing the gap between frames and events. Exponential decays can be used to convert events to frames compatible with such screens.
