# Supplementary material for: Sepia, Tarsier, and Chameleon: A Modular C++ Framework for Event-Based Computer Vision
Source: Front Neurosci. 2020 Jan 8;13:1338. doi: 10.3389/fnins.2019.01338 (PMC6960268; doi:10.3389/fnins.2019.01338)
Supplement: Supplementary file 1 [file Data_Sheet_1.PDF]

Sepia, Tarsier and Chameleon: a modular C++ framework for event-based computer vision

Alexandre Marcireau [1], **Sio-Hoi Ieng** [1*], **and Ryad Benosman** [1,2,3]
*Correspondence:
Sio-Hoi Ieng
siohoi.ieng@gmail.com

APPENDIX

1 FILE FORMAT

1.1 Containers

Multiple file formats co-exist to represent events generated by a camera. This appendix presents considerations to improve over these formats, and makes the first step in the suggested direction with a novel specification. This specification is used by the *sepia* library.

The C++ frameworks mentioned in this paper use the *dat* file format (*kAER*) or the *aerdat* format (*jAER*, *cAER* and *Dynamic Vision Systems*). The *dat* format has no official specification, and thus is hard to use without Prophesee's proprietary libraries. The *aerdat* fornat uses packets mixing frames and events, reflecting the output format of the DAVIS. Packets make the generation of artificial data more complex, since encapsulation in packets of arbitrary length is mandatory. Moreover, the format does not benefit from state-of-the-art frame-based encoding schemes, such as HEVC or VP9 (**?**).

Conventional multimedia files use containers, defined as a format wrapper holding common meta-data (title, author, creation date…) and encapsulating data streams. Each stream is encoded in a data format describing a specific type of information. This organization is modular and simplifies the design of data formats, as they do not need to account for meta-data.

A container for event-based data would wrap - among others - streams of visual events, IMU events, cochlea events and frames. The latter would take advantage of existing formats for frame-based data. Potential compression algorithms for event streams would progressively replace current events encoding without changing the container format.

We introduce a simple, well-defined data format called *Event Stream*, with extension **.es**. The format encodes only visual events, without packets. It cannot encode meta-data, apart from information required for proper decoding and processing (namely, the format version and the sensor width and height). Even though this data format can be used alone, it is meant to be included in a container format (which does not exist yet).

1.2 Event Stream

The data associated with a visual event can be represented as a tuple (x, y, t, p), where x and y are the pixel coordinates, t is the timestamp (generally expressed in microseconds) and p is the polarity. The latter has an arbitrary size in the general case, and is a boolean for *DVS*-like polarity events.

Since the events are ordered, t increases from one event to the next, often by a small value. This property is leveraged by the *aerdat* format: the timestamp is encoded relatively to the packet beginning, reducing the number of bits required to encode it. Using a similar approach, a packet-free scheme can be devised:

- An event is encoded by k timestamp bits and 33 payload bits (16 bits for x, 16 bits for y and 1 bit for the polarity). Only timestamps in the range $[\![0, 2^k - 2]\!]$ are used.
- The special value $2^k - 1$ encodes a timestamp overflow: the decoding program must increment by $2^k - 1$ an offset variable initialized to zero and added to every timestamp. The 33 payload bits are not included in this case.

We refer to this scheme as absolute encoding. A variant, called relative encoding, consists in encoding each timestamps relatively to the previous one. The offset variable must be incremented with every event. Relative timestamp overflows are handled similarly to absolute timestamp overflows.

The throughput of the absolute and relative encoding schemes depends on k and the event stream content. Figure S1 (top) shows the throughput of various streams as a function of k. The previously described *car* and *street* streams (table 2), as well as the initial second of the *street* stream, are used. The beginning of the *street* stream contains very little activity (the average even rate is $22.2 \times 10^3\,\mathrm{s}^{-1}$), illustrating the schemes behaviour in this situation. The minimum throughput is obtained for surprisingly small values of k. Notably, the optimal value for k is one for the *car* stream. With this k, both schemes are identical and are equivalent to writing a binary one every time the clock advances by one microsecond, and a zero followed by the payload for each event. The performance variation between schemes is small compared to the throughput. Nevertheless, the relative scheme outperforms the absolute one. The difference increases with the activity, yielding a better compression when it is most needed.

The absolute encoding is sensitive to bit errors: they can create non-monotonic timestamps, thus negative time deltas. The latter are used in many algorithms, and negative values generally result in unexpected behaviors. Bit errors are less serious for the relative encoding: they result in small time distortions. Consequently, the *Event Stream* format specification is based on the relative scheme.

The schemes considered so far use a non-round number of bytes to encode each event. This approach complicates the implementation, since bytes are the fundamental type of most operating systems. Therefore, the *Event Stream* specification uses $k = 7$ (even though it is not the optimal value for high-activity streams) so that each event is encoded on 5 bytes. Offsets are encoded on one byte. The last bit is used to differentiate overflow bytes and reset bytes. The latter must be sent periodically if the encoding is used in a noisy environment. Upon reception, they reset the state machine illustrated figure S2.

The *Event Stream* specification also supports ATIS events, color events and generic events (with an arbitrary payload associated with each timestamp). It is designed to be extended.

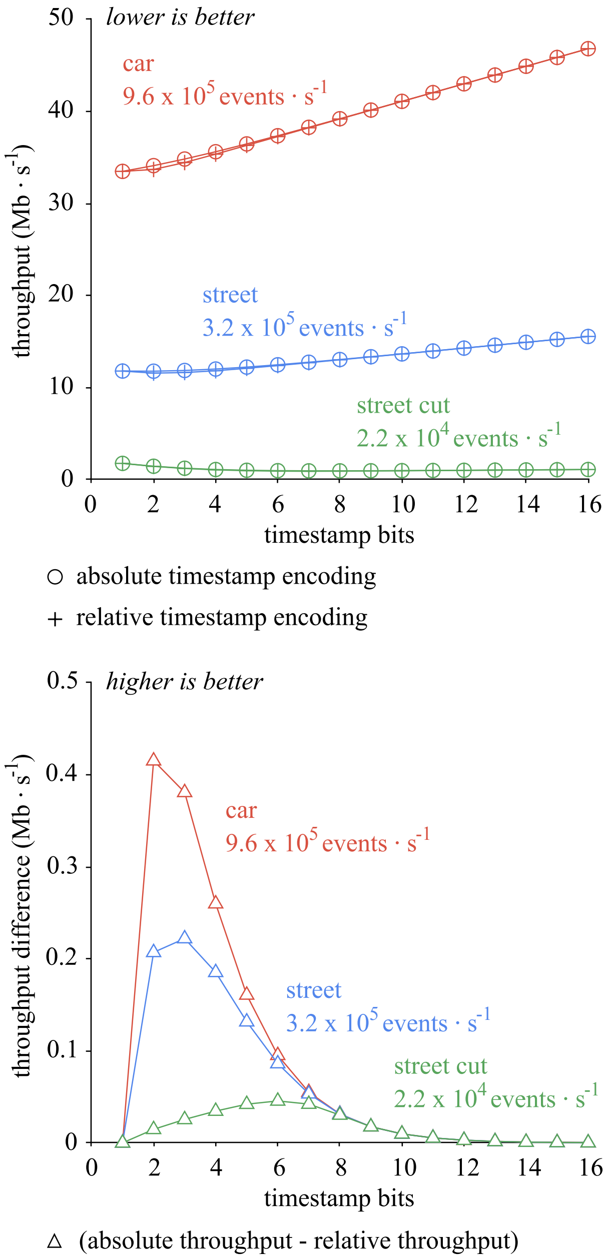

Figure S1. The top graph plots the throughput (the number of bits required to encode the stream) as a function of the number of bits used to encode the timestamp. The absolute scheme is represented with circles, and the relative scheme with crosses. The bottom graph shows the throughput difference between schemes.

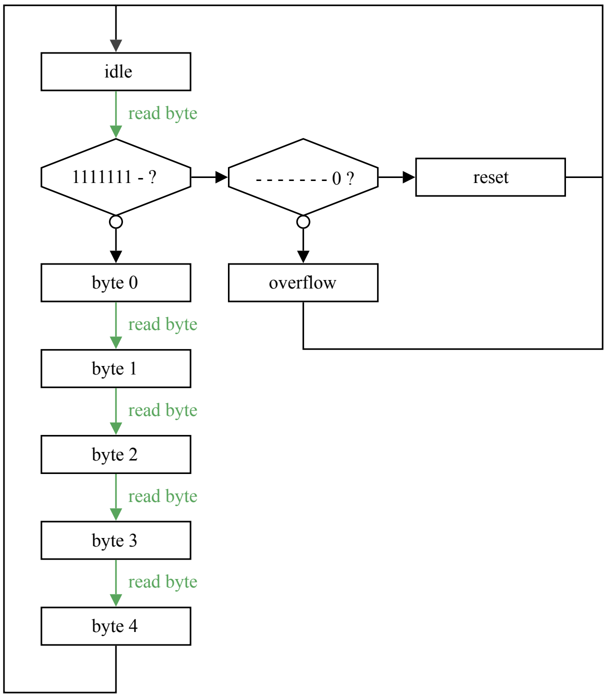

Figure S2. This state machine describes the *Event Stream* specification for *DVS*-like events. The format is designed for file IO, but can be used over any serial communication channel, such as USB.
